# Performance of Indian Children with Cochlear Implant on PEACH Scale

**DOI:** 10.1155/2013/565096

**Published:** 2013-03-04

**Authors:** Suman Kumar, Nachiketa Rout, Navnit Kumar, Indranil Chatterjee, H. Selvakumaran

**Affiliations:** ^1^Department of Speech and Hearing, ERC, AYJNIHH, B.T. Road, Bon Hooghly, Kolkata 700090, India; ^2^NIEPMD, East Coast Road, Kovalam Post, Muttukadu, Tamil Nadu, Chennai 603112, India; ^3^All India Institute of Speech & Hearing (AIISH), Manasagangothri, Mysore 57000, India; ^4^Astra Hearing Care Centre, Unit No. 5, Iswarya Laxmi Plaza, K. K. Nagar, Tamil Nadu, Madurai 625020, India

## Abstract

This study compares the functional language performance of Tamil-speaking children (*n* = 30) who received a cochlear implant (CI) before 2 years of age (earlier implanted group: EIG) and between 3 and 4 years of age (later implanted group: LIG). Everyday functional language of children was evaluated by interviewing parents using the adapted Parents' Evaluation of Aural/Oral Performance of Children (PEACH) Questionnaire in Tamil language. On average, both groups of children had difficulties in everyday language functioning. However, functional results of EIG were better than those of LIG. In addition significant correlations were found between age at intervention and PEACH score. The evidence lends support to early intervention increasing the functional performance of the children fitted with CI. PEACH can be a clinically feasible evaluation tool to implement in practice for clinicians to obtain meaningful information regarding children's auditory performance in real life at childhood.

## 1. Introduction

Young children with severe to profound sensorineural hearing loss face challenges in developing spoken language, literacy, psychological functioning, and academic achievement [1–4]. Parents, educators, clinicians, and researchers also agreed that language acquisition in young children with severe to profound hearing loss represents a major challenging issue for them [5]. They reported that children with permanent hearing loss are unable to detect acoustic-phonetic cues essential for speech recognition, even when fitted with traditional amplification devices [4]. Studies reported that, in these children, cochlear implant (CI) provides significant gain in auditory perception and speech production [6, 7]. Osberger [8] claimed that the cochlear implant has a dramatic impact on improving the acquisition and use of spoken language by deaf children, with positive ripple effects socially and psychologically. Therefore improvement in speech and language skills has been considered as an essential goal in children having cochlear implantation [9, 10]. 

Although the auditory information provided by CI is not as rich and complex as normal hearing [11], prelinguistically deaf children who receive a cochlear implant before the age of 10 years gained significantly better speech production skills than children who implanted later [12]. Furthermore, they learned language at a faster rate than normal peers [4, 13]. Studies also reported that children with CI attained language levels near to similar-age peers with normal hearing after up to 5 years of implant use [14, 15]. 

Most of the studies to date used standardized language tests to assess the language performance in children with CI [16–19]. The commonly used are Preschool Language Scale version 4 [20], Peabody Picture Vocabulary Test version 4 [21], Diagnostic Evaluation of Articulation and Phonology [22], and Clinical Evaluation of Language Fundamental [23]. These tests provide child's performance as a standard score in relation to normative samples of age-matched hearing children. It evaluates (a) the extent of linguistic abilities acquired, (b) different components of language achieved: receptive and expressive abilities, and (c) vocabulary and grammar. These are considered as reliable indicators of child's linguistic achievement [24]. However, it is still unclear that to what extent the language ability assessed in a structured setting reflects the ability of children to function in everyday life [17]. Thus formal language measures do not reflect the realistic picture of functional language performance, that is, language ability of children to function in everyday life. This may be because children with CIs have different levels of proficiency on the different language domain [18, 19]. Furthermore, Duchesne et al. [5] concluded from a systematic review on the language development in children who received CI below 3 years of age their magnitude of language improvement may not be uniform across language components. However, differential performance can be best quantified using standardized language tools. But realistic picture of differential proficiency on language domains of children with CI can be seen only by assessing the language performance in different environments with different people [25].

Most of the studies to date examined language development on a group of children who received their implants by the age of 36 months or slightly above [26–30]. Few studies have examined language development on a group of children who underwent cochlear implantation around the age of 2. Functional language performance is difficult to assess in young infants and toddlers with hearing impairment due to their immature developmental level and language abilities. Thus researchers rely on parent report to ascertain functional performance in these populations to assess spontaneous responses to sound in everyday environment. The commonly administered parental report scales are Meaningful Auditory Integration Scale (MAIS [31]), Infant Toddler Meaningful Auditory Integration Scale (IT-MAIS [32]), LittlEARS Auditory Questionnaire (LEAQ [33]), and The Parents' Evaluation of Aural/Oral Performance of Children (PEACH) scale [16]. Amongst these, PEACH scale is commonly used with children from any age group and with hearing loss ranging from a mild to a profound degree [34]. The scale needs to rate the presence and absence of listening skills as well as to write down examples of the auditory behavior of their children in day-to-day life in response to each of the items. Thus it requires an active participation of parents to observe their child in real context and provides an opportunity to report their observations freely instead of restricting their answers to the test agenda [35]. Ching et al. [17] assessed language ability and everyday functioning of 133 children with hearing impairment. They were evaluated at 3 years of age. The language abilities were evaluated using the Preschool Language Scale (PLS-4), Peabody Picture Vocabulary Test (PPVT), Diagnostic Evaluation of Articulation and Phonology (DEAP), and Child Development Inventory (CDI). Everyday functioning of children was evaluated by interviewing parents using the PEACH questionnaire. They reported a significant correlation among language measures and also between the standardized language measures and the PEACH. On average, children who had language deficits exhibited difficulties in everyday functioning. They suggested use of PEACH scale to evaluate young children's aural/oral communicative functioning in everyday life. This scale is considered as a reliable measure for evaluating the effectiveness of amplification for children in real life [36].

In account of the above view, this study aimed at comparing the effect of age at intervention of children fitted with cochlear implants on everyday functional language performance in different situations. The functional language performance was assessed using adapted PEACH in Tamil language. Tamil has an official status in the Indian state of Tamil Nadu and in the Indian union territory of Puducherry. This is also an official language of Sri Lanka and Singapore. The study also looks into the correlation between age at cochlear implantation and Tamil PEACH scale scores.

## 2. Method

### 2.1. Participants

Thirty parents/primary caregivers of children with cochlear implant, 3–6 years old, were included in this study. Participants were grouped according to age at cochlear implantation. Earlier implanted group (EIG) was implanted before 24 months (*M* = 17.2, SD = 1.2). However, later implanted group (LIG) was implanted between 36–48 months (*M* = 41.3, SD = 1.4) of age. Although both groups have equal number of participants (*N* = 15), they vary in gender. EIG consisted of eleven males and four females within the age range of 22–32 months (*M* = 29.2, SD= 2.1). However, LIG had ten males and five females within the age range of 49–57 months (*M* = 53.3, SD = 1.1). 

All the participants had severe to profound sensori neural hearing loss. The hearing loss was identified before their first birthday (*M* = 6.4, SD = 1.6). None of the participants fall under the Joint Committee of Infant Hearing [37] risk factors for hearing loss. Thus it has been assumed that they all had congenital hearing loss. Apart from that, motor developmental milestones achieved age appropriately. The intervention (hearing aid fitting and AVT) started within four months (*M* = 3.3, SD = 1.3) after identification of hearing loss. Prior to CI, binaural behind the ear (BTE) along with AVT (45-minute session, 5 times a week) in Tamil had been experienced for a minimum of six months. Afterward, EIG and LIG underwent cochlear implantation within five months (*M* = 3.8, SD = 1.4) and 20–32 months (*M* = 28.7, SD = 1.5), respectively. Both groups were fitted with monaural ESPrit Nucleus device with advance combinational encoder strategy. Along with cochlear implantation, they also attended AVT (45-minute session, 5 times a week) at MERF, Chennai, India. They experienced the cochlear implant along with AVT for a minimum of one year. In addition, all participants belonged to middle-class socioeconomic status. Parents/primary caregivers had an education level higher than 9 years in Tamil medium. Mean schooling year was 11.08 (SD = 3.6).

### 2.2. Procedures

#### 2.2.1. Test Adaptation

PEACH scale was translated and adapted into Tamil language with the help of an audiologist and a linguist. They reviewed the available literature in Tamil language from books, journals, web-based sources, and existing tools in India. The questionnaires in Tamil version were seen for syntactic structure, semanticity, familiarity, and ambiguity. Therefore the original meaning and concepts of the questionnaires were unchanged and also culturally appropriate. The translated and modified material was judged by five experienced audiologists. It was rated on a “Feedback Questionnaire for Aphasia Treatment Manuals” [38], which includes rating ranging from very poor to excellent on given 17 parameters except 3 parameters (volume, size of the picture, and color of the picture).

#### 2.2.2. Test Administration

The adapted items were administered on participants. Participation in this study was voluntary. At first participants signed an informed consent form. This document aimed at informing the participants about the objectives, justifications, and procedures of this investigation. The adapted version followed similar guidelines to PEACH scale described by Ching and Hill [16]. Including the first author two audiologists administered the adapted PEACH. Both of them were native Tamil speakers and were trained in the procedure of administering the PEACH scale. The PEACH includes 13 questionnaires that assess (a) use of amplification and loudness discomfort, (c) listening and communicating in quiet and noise, (d) use of telephone, and (e) responses to environmental sounds.

The Tamil PEACH was provided to the parents/primary caregivers. Each question was explained by the authors. An interview session was arranged to clarify any doubts or suggestion. Afterwards they were asked to observe the auditory and oral behavior of their children in relation to each question for a period of two weeks. They were also instructed to note down the as many examples of responses for each question and videorecord the situation if possible. After completion of test items, another interview session was arranged with parents/caregivers to clarify the recorded unclear examples of response. The clarification will help to increase the accuracy of response behavior.

#### 2.2.3. Scoring

The first researchers scored each response of the question based on the information provided by parents/caregivers. They used the scoring protocol of Ching and Hill [16]. Each response to a question was scored on a five-point rating scale ranging from 0 to 4. The descriptive criteria for rating was as follows.

(0) No examples were given or child did not demonstrate any auditory response.
(1) If one or two examples were provided or auditory response occurred 25% of the time.
(2) If three or four examples were provided or auditory response occurred 50% of the time.
(3) If four or five examples were provided or auditory response occurred 75% of the time.
(4) If more than six examples were provided or response occurred more than 75% of the time.

#### 2.2.4. Test-Retest Reliability

During the last interview session parents were asked to participate in the repeatability of the test if interested. Six parents/caregivers participated for the second time. Again periods of two weeks were provided to observe the responses.

#### 2.2.5. Analysis

All data were recorded into Statistical Program for Social Sciences (SPSS) 16.0 for statistical analysis. Mean PEACH scores of both EIG and LIG were compared. Further, chi-square test was utilized to evaluate statistical differences between the categorical data. The Spearman rank correlation was used to evaluate the linear association between age of cochlear implantation and PEACH scores. Test-retest reliability for ratings was calculated using correlation analysis.

## 3. Results

### 3.1. Comparison of Tamil PEACH Scores between EIG and LIG

**Figure 1:**
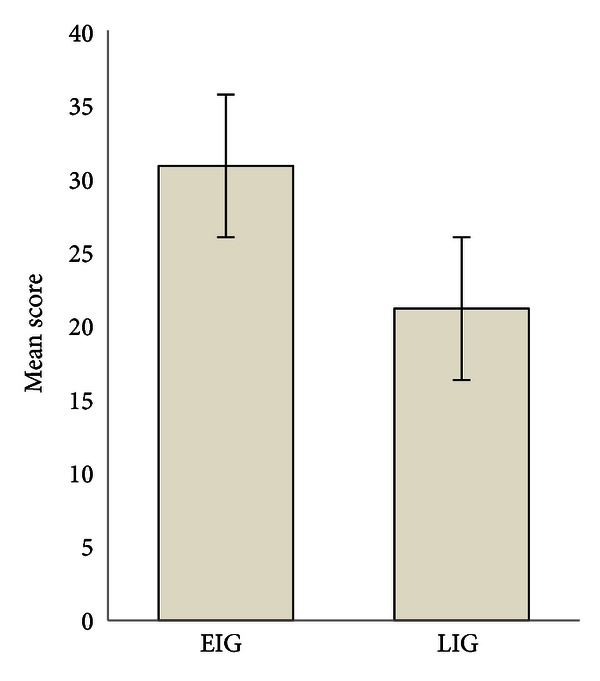
Mean Tamil PEACH scores as a function of intervention groups (EIG and LIG). Error bars show SD.

Table 1 shows the mean PEACH score, range, and standard deviation (SD) for both groups. EIG obtained higher PEACH score than LIG. The PEACH scores obtained in both groups are shown in Figure 1. The standard deviations obtained from both groups are less than one, suggesting low variability of individual PEACH score. When analysis was performed, significant difference was found between the Tamil PEACH score of both groups (*t*(29) = 17.03, *P* = 0.033). However, EIG and LIG show high negative (Spearman rank correlation coefficient = −0.76, *P* < 0.05) and low negative (Spearman rank correlation coefficient = −0.55, *P* < 0.05) correlation with Tamil PEACH score, respectively.

### 3.2. Test-Retest Reliability

The Tamil PEACH was administered twice on six parents (EIG = 4; LIG = 2). The mean score differences were much less than the overall score.  Table 2 shows the mean score difference obtained from parents after twice test Tamil PEACH administration.

## 4. Discussion

The present study used outcome measures designed to compare the everyday functional language performance of children who received cochlear implant before 2 years of age (EIG) with children who received implants between ages 3 and 4 years (LIG). The performance was assessed using adapted PEACH (Tamil). Tamil PEACH was administered to parents of Tamil children and follows the same procedure as used by Quar et al. [34]. Test-retest reliability was obtained from six participants when the adapted scale was administered twice. The results indicate high test-retest reliability. The results (means) reveal that children who received implants before 2 years showed significant higher language ability to function in daily life as compared to those received at 36–48 months Although the present study did not measure the language score of children using standardized language tools, the study reported that children's everyday language functioning as observed by PEACH scores significantly correlates with language ability measured using standardized language tools [17].

Developmental studies signify the importance of critical or sensitive period for development of linguistic structure [39, 40]. During critical period the developing central nervous system can most readily use sensory information to form linguistic structure. However, it may vary according to the different elements of language. For example, 6 months of fetal life through the age of 12 months is critical for phonetic factors, up to the age of 4 years is for syntax, and up to the age of 16 years for semantics [41]. In addition to the possible existence of critical periods for language learning, there is a complementary argument supporting the importance of young age at implantation. In the present study, variables like amount of hearing loss, types of implant, and socioeconomic status were controlled, except for age of intervention. Age of intervention was negatively correlated with PEACH scores. It means as age progressed, the functional language performance decreased. Thus children who received CI at three years of age or higher may experience difficulties in everyday functioning in different situations. In support of these findings, Ching et al. [17] found that children who received intervention before 6 months of age attained language levels below 1 SD of the normative mean on several standardized measures. The finding suggests that children experienced disadvantages in language development and everyday functioning at a young age even when they were detected and intervened early. On the other hand, studies also reported that language development after cochlear implantation before the age of 3 showed that some children with CIs appear to learn language at a normal or near-normal rate, allowing the gap between language age and chronological age to narrow, or at least to remain constant [30, 42–44]. Novak et al. [45] found that children who received a CI between 9 and 25 months of age obtained language scores equal to or above chronological age than who received the implant between 20 and 25 months. Manrique et al. [46] found that children who received a CI by the age of 2 years showed an expressive delay of approximately 1 year despite a normal growth rate, whereas a greater expressive delay was found in children who received a CI after the age of 2. In addition, children with CIs may have learned language at a faster rate than normal, thus enabling them to demonstrate language levels on par with their same-age hearing peers after up to 5 years of implant use [15]. Contrary to the above findings, EIG in the present study did not obtain maximum score (44). This may be because of only one year of implant experience. At the same time, functional language performance score of EIG was not compared with age-matched normal peers. However it is clear that early auditory experience with CI provides an advantage to children with a better spoken language performance than older age at implant.

Although a number of factors other than age of intervention might influence the functional language performance. Parental/caregivers' involvement in early communication is also associated with spoken language performances [47]. In early life, a parent-child interaction in natural communication environment influences the comprehension and expression of a child. The early interaction provides a cue for language learning and neuronal development. Therefore, language exposure and proper monitoring by caregivers provide the context for language learning in early developmental stages. Although language exposure was not quantified in the present study, medical history reported by the parents revealed no particular medical condition that might affect functional language performance. 

Pungello et al. [48] investigated the effects of socioeconomic status, race, and parenting on early childhood auditory/oral skills. They found that African-American children obtained lower scores for receptive and expressive language when compared with European-American children. Thus parenting style (maternal sensitivity and negative intrusive maternal behavior), maternal education level, family stress, race, parent-child interaction, and cultural difference can affect language development of children [34]. Previous studies reported that Chinese parents, when compared to American and Canadian parents, are more restrictive, controlling, or authoritarian and less affectionate [49–51]. Thus it was needed to study the Asian children to examine the impact of parenting and cultural differences on children's functional language performance.

The present study utilizes the adapted PEACH Tamil. Further work needs to establish norms for a larger sample of children with normal hearing and for children using CI. These normative data for children using CI in the Tamil-speaking environment will be useful for clinical applications. It will be helpful to compare the performance of children with CI to their normally-hearing peers. 

## 5. Conclusion

CI before 2 years of age provides better functional language performance than older age at implant [52]. Considering significant correlation of PEACH scores with language ability measured using standardized language tools, it can be used to a population where standardized tools cannot be easily administered. Thus in an early period of life they are very useful tools for clinicians to obtain meaningful information regarding children's auditory performance in real life. In addition PEACH measure is also useful in evaluating functional language performance of children whose primary mode of communication is not English. 

## Figures and Tables

**Table 1:** Mean PEACH scores, standard deviation (SD), and range.

Groups	Mean (SD)	Range
EIG (*N* = 15)	30.8 (.98)	28–38
LIG (*N* = 15)	21.13 (.27)	16–27

**Table 2:** Mean test-retest difference score and standard deviation.

Groups	1st mean score (SD)	2nd mean score (SD)	Mean score difference
ELI	30.8 (.98)	32.1 (.65)	1.3 (.51)
LIG	21.13 (.27)	23.4 (.78)	2.2 (.26)
